# Resting state fMRI analysis of pseudobulbar affect in Amyotrophic Lateral Sclerosis (ALS): motor dysfunction of emotional expression

**DOI:** 10.1007/s11682-022-00744-4

**Published:** 2022-11-12

**Authors:** Francesca Trojsi, Federica Di Nardo, Giulia D’Alvano, Giuseppina Caiazzo, Carla Passaniti, Antonella Mangione, Minoo Sharbafshaaer, Antonio Russo, Marcello Silvestro, Mattia Siciliano, Mario Cirillo, Gioacchino Tedeschi, Fabrizio Esposito

**Affiliations:** Department of Advanced Medical and Surgical Sciences, MRI Research Center, Università degli Studi della Campania “Luigi Vanvitelli”, P.Zza Miraglia 2, 80138 Naples, Italy

**Keywords:** Amyotrophic lateral sclerosis, Resting state functional MRI, Pseudobulbar affect, Pathological laughing and crying, Cerebellum

## Abstract

Pseudobulbar affect (PBA), referring to exaggerated or inappropriate episodes of laughing and/or crying without an apparent motivating stimulus, has been mainly attributed to bilateral degeneration of corticobulbar tracts. We aimed at exploring brain functional connectivity (FC) correlates of PBA in patients with amyotrophic lateral sclerosis (ALS), the most common motor neuron disease, frequently associated with PBA. Resting state functional MRI (RS-fMRI) independent component (ICA) and seed-based analyses and voxel-based morphometry (VBM) whole-brain analysis were performed on 27 ALS patients (13 with PBA; 14 without PBA) and 26 healthy controls (HC), for investigating functional and structural abnormalities in ALS patients compared to HC and in patients with PBA compared to patients without PBA. Between-patient analysis revealed different FC patterns, especially regarding decreased FC in several areas of cognitive (default mode, frontoparietal, salience) and sensory-motor networks in patients with PBA compared to those without PBA. However, no significant differences were found in gray matter atrophy. Seed-based analysis showed increased FC between middle cerebellar peduncles and posterior cingulate cortex and decreased FC between middle cerebellar peduncles and left middle frontal gyrus in patients with PBA compared to patients without PBA. Our findings suggest that some alterations of fronto-tempo-parietal-cerebellar circuits could be related to PBA in ALS. In particular, the abnormal FC between cerebellum and posterior cingulate cortex and left middle frontal gyrus in patients with PBA compared to patients without PBA highlights a crucial role of the cerebellum in regulating emotion expression in patients with ALS.

## Introduction

Pseudobulbar affect (PBA) is characterized by involuntary and uncontrollable outbreaks of laughing and/or crying (pathological laughing and crying, PLC) that are incongruous or disproportionate to the patient’s emotional state (Dark et al., 1996). Moreover, pathological yawning, more frequently described in disorders affecting the brainstem, may also occur in supratentorial alterations, including PBA in stroke (Singer et al., 2007). This condition may arise from the disconnection of brainstem structures from cortical inhibition and can occur in several neurological diseases, such as strokes, traumatic brain injuries, Alzheimer’s disease, multiple sclerosis, amyotrophic lateral sclerosis (ALS), and primary lateral sclerosis (PLS) (Finegan et al., 2019, 2021; Parvizi et al., 2006, 2009; Work et al., 2011). At the population level, up to 50% of patients with motor neuron diseases, and in particular those with bulbar upper motor neuron (UMN) involvement, are affected by this condition, and one-third of ALS patients present PBA at diagnosis (Tortelli et al., 2016; Hübers et al., 2016which represents a negative prognostic symptom (Barć et al., 2020; Tortelli et al., 2018). However, although PBA is a frequent and long-reported symptom, it has remained unclear if this phenomenon is a result of a lack of inhibition from the frontal cortex ("top-down-theory") or due to altered processing of sensory inputs at the brainstem level ("bottom-up-theory") (Bede & Finegan, 2018). Wilson (1924) hypothesized that exaggerated or involuntary emotional expression was caused by opercular and corticopontine tract degeneration. These lesions were supposed to result in a loss of voluntary control of functional brain stem regions that in turn drive emotional expression. The second, more recent theory claims that the brainstem response itself is impaired in patients with PBA, which in turn generates a pathological and exaggerated reaction to sensory stimuli (Parvizi et al., 2009). Dysfunction of cortico-ponto-cerebellar networks is regarded as a key factor in exhibiting context-inappropriate emotional responses (Lapchak, 2015). This theory was confirmed by observations of different lesions such as those related to strokes (Lapchak, 2015; Wang et al., 2016), brain prematurity (Bodensteiner, 2018), or tumors (Pollack et al., 1995) in the pons, cerebellum, and brainstem. However, although PBA is a common feature in ALS, only a few studies exist in this patient group with the aim of targeting this phenomenon and the brain circuits underpinning it. In this regard, advanced neuroimaging techniques may offer a unique opportunity to clarify the contribution of specific neural circuits to the etiology of PBA.

In the last decade, a diffusion tensor imaging (DTI) study in ALS patients with PBA showed disruption of fiber tracts descending from the fronto-temporal cortex towards the pons and degeneration in the transverse pontine fibers and middle cerebellar peduncles (Floeter et al., 2014). Christidi et al. (2018) identified widespread orbitofrontal, opercular, and putaminal grey matter (GM) alterations in ALS patients with pathological crying and laughing, also mapping white matter (WM) changes to the posterior cingulum, posterior corona radiata, superior corticospinal tracts, cerebellar peduncles, and fornix. Tu et al. (2021) recently emphasized the importance of WM and GM brainstem integrity in the development of pathological laughter. In addition, Klingbeil et al. (2021), applying a "lesion network-symptom-mapping" to focal lesions identified in a systematic literature search for case reports of PLC, identified an emotional system that exerts excitatory control of the periaqueductal grey (descending from the temporal and frontal lobes, basal ganglia, and hypothalamus), and a volitional system (descending from the lateral premotor cortices) that can suppress laughter or cry. However, no investigation of functional connectivity (FC) abnormalities has been performed in ALS patients with or without PBA to explore resting state functional MRI (RS-fMRI) networks (RSNs) alterations underlying this syndrome.

Based on these considerations, our main objective was to shed more light on the cerebral substrates of PBA in ALS and to increase previous MRI findings on this topic by addressing the RS-fMRI profile of connectivity dysfunction associated with PBA. We investigated the FC impairment of brain networks by performing the independent-component analysis (ICA) and seed-based RS-fMRI analysis, while an investigation of whole-brain GM abnormalities was performed through voxel-based morphometry (VBM) analysis in non-demented ALS patients with or without PBA compared to HC. Our main hypothesis was that different RS-fMRI patterns of abnormalities could be revealed in the two groups of patients compared with each other.

## Methods

### Case selection

Twenty-seven right-handed patients (23 males; mean age 58 ± 12), with definite or clinical/laboratory-supported probable ALS, according to the El-Escorial revised criteria (Brooks et al., 2000), showing classic, bulbar, flail limbs or pyramidal phenotypes (Chiò et al., 2011), revealed to be able to tolerate the MRI exam, were consecutively recruited at the First Division of Neurology of the University of Campania “Luigi Vanvitelli” (Naples, Italy) from January 2020 to July 2021.

As for clinical features, we measured: disease duration (from symptom onset to scan date in months); ALSFRS-R total score (0–48, with lower total reflecting higher disability), and subscores (i.e., bulbar, fine-motor, gross-motor, and respiratory subscores) (Cedarbaum et al., 1999); and upper motor neuron (UMN) score, index of pyramidal dysfunction through the evaluation of the number of pathologic reflexes elicited from 15 body sites (Turner et al., 2004). ALS patients underwent the Italian version of the Edinburgh Cognitive and Behavioural ALS Screen (ECAS) (Poletti et al., 2016; Siciliano et al., 2017) for assessing global cognitive functioning, and the Italian version of the Center for Neurologic Study-Lability Scale (CNS-LS) for assessing PBA, validated in ALS (Moore et al., 1997). CNS-LS is a 7-item self-administered questionnaire. Each item asks respondents to indicate on a 5-point scale how often they experience the symptom (1 = applies never; 5 = applies most of the time) over the last week. Total scores range from 7 to 35. Pseudobulbar (PSB) patients had a CNS-LS score ≥ 13. Moreover, depressive symptoms were measured by Beck Depression Inventory-II (Beck et al., 1996). Cut-off score of 20 points for BDI-II was adopted for revealing the presence of clinically significant depressive symptoms.

Genetic analysis was performed in all patients, exploring *C9orf72* repeat expansion and mutations of *SOD1*, *TARDBP,* and *FUS/TLS*. No mutations of these genes were reported.

Twenty-six right-handed HC (20 males; mean age 58 ± 12) were enrolled by "word of mouth" and among caregivers’ friends. They were age-, sex-, and education-matched with the enrolled ALS patients. Their Mini-Mental State Examination (MMSE) scores (Folstein et al, 1975) were ≥ 27. For all subjects, exclusion criteria included: medical illnesses or substance abuse that could interfere with cognitive functioning; any other major systemic, psychiatric, or neurological diseases; other causes of brain damage, such as lacunae and extensive cerebrovascular disorders at MRI; and a vital capacity of less than 70% of the predicted value (to avoid bias from respiratory compromise on cognitive measures).

### Statistical analysis: between-groups comparisons of clinical data

Shapiro–Wilk tests were used to assess normality and, according to the distribution of the data, t-tests and Chi-square tests were used to compare demographics and neuropsychological scores between ALS patients and HC. For all analyses, IBM SPSS v. 25.0 was used, and the level of significance was set at p.05.

### MRI analysis

#### Magnetic resonance imaging

MRI images were acquired on a 3 T scanner equipped with a 32-channel parallel head coil (General Electric Healthcare, Milwaukee, Wisconsin). The imaging protocol included three-dimensional T1-weighted images (gradient-echo sequence). Inversion Recovery prepared by Fast Spoiled Gradient Recalled-echo, repetition time = 6.988 ms, inversion time = 650 ms, echo time = 3.0 ms, flip angle = 9, voxel size = 1 × 1 × 1 mm3); RS-fMRI sequence, consisting of 320 volumes of a repeated gradient-echo echo-planar imaging T2*-weighted sequence (repetition time = 1500 ms, echo time = 19 ms, nr. of axial slices = 44, matrix = 96 × 96, field of view = 288 mm, thickness = 3 mm, interslice gap = 0 mm, voxel size = 3 × 3 × 3 mm3); T2-fluid attenuation inversion recovery to exclude severe cerebrovascular disease according to standard clinical neuroradiological criteria on visual inspection by three experienced radiologists. During the functional scan, subjects were asked to simply stay motionless, awake, and relaxed and to keep their eyes closed. The total scanning time for RS-fMRI was set to about 8 min, so the chance of falling asleep would be much reduced. Immediately after the scan, each participant was asked questions to verify their degree of cooperation. Anyway, all ALS patients had a normal respiratory function and did not suffer from specific sleep problems. No visual or auditory stimuli were presented at any time during functional scanning. The total duration of the imaging session was about 38 min.

#### RS-fMRI data preparation and preprocessing

Standard functional image data preparation and preprocessing, statistical analysis, and visualization were performed with the software Brain Voyager QX (Brain Innovation BV, Maastricht, The Netherlands). Data preprocessing included the correction for slice scan timing acquisition, a three-dimensional rigid-body motion correction based on a 6-parameter rigid-body alignment to correct for minor head movements, the application of a temporal high pass filter with a cut-off set to 0.008 Hz, and spatial smoothing of image series with a 6 mm full-width at half-maximum isotropic Gaussian kernel. Translational motion parameters were verified to always be less than 1 functional voxel for all included participants. The mean frame-wise displacement (FD) (i.e., a surrogate metric of head motion accounting for intra-voxel residual motion effects) was also estimated from the translational and rotational parameters, and a typical cut-off of 0.5 mm was applied (Power et al., 2014). We further verified that there were no statistically significant differences in the mean FD when carrying out group comparisons. Structural and functional data were coregistered and spatially normalized to the Talairach standard space using a 12-parameter affine transformation.

#### Resting State Network (RSN) functional connectivity analysis

To extract RSN maps, single-subject, and group-level ICA were carried out on the preprocessed functional time series using 2 plug-in extensions of Brain Voyager QX (Goebel et al., 2006), respectively, implementing the fast ICA algorithm (Hyvärinen et al., 2001) and the self-organizing group ICA algorithm (Esposito et al., 2005). Furthermore, the ICASSO procedure (Himberg et al., 2004) was applied to the extraction of individual ICA components, according to previously described methods (Trojsi et al., 2021).

For every single subject, 50 independent components were extracted (corresponding to ~ 1/6th of the number of time points) (Greicius et al., 2007) and scaled to spatial z scores (i.e., the number of standard deviations of their whole-brain spatial distribution). To generate group components and allow for group-level inferences in each RSN, all individual component maps from all subjects were "clustered" in the subject space according to the mutual similarities of their whole-brain distributions using the self-organizing group ICA algorithm. Therefore, all 50 individual independent components were uniquely assigned to 1 out of 50 "clusters" of independent components. Once the components belonging to a cluster were selected, the corresponding maps were averaged, and the resulting group map was taken as the representative FC pattern of the cluster. The 50 single-group average maps were visually inspected to recognize the spatial patterns associated with the main RSNs (Smith et al., 2009). For this purpose, single-group 1-sample t-tests were used to analyze the whole-brain distribution of the components in each group separately, and the resulting t maps were thresholded at *p* = 0.05 (Bonferroni corrected over the entire brain) after regressing out age and gender from the series of individual maps at each voxel. An inclusive mask was finally created from the healthy control group maps and used to define the search volume for within-network 2-group comparisons. These comparisons were performed by fitting a one-way analysis of variance (ANOVA) model that included one between-subjects factor with three levels: pseudobulbar (PSB) ALS patients, non-pseudobulbar (nPSB) ALS patients, and HC, and then calculating post hoc t contrasts for obtaining between-group t maps. To correct the resulting t maps for multiple comparisons, regional effects within the search volume were only considered significant for compact clusters emerging from the joint application of a voxel-level and a cluster-level threshold. The cluster-level threshold was estimated non-parametrically with a randomization approach: we calculated the FWHM from each RSN t map for the HC group and then, starting from an initial (uncorrected) threshold of *p* = 0.001 applied to all voxels, a minimum cluster size was calculated that protected against false-positive clusters at 5% after 1000 Montecarlo simulations (Forman et al., 1995).

#### Seed-based connectivity analysis

A seed-based analysis was performed to study FC between specific regions of interest, including right and left pre-central gyri, right and left middle cerebellar peduncles, and pons, as identified according to previous studies of the brain circuit underpinning PBA (Floeter et al., 2014; Parvizi et al., 2001), and the entire rest of the brain. For this purpose, nuisance signals [global signal, WM, and cerebrospinal fluid (CSF) signals] were regressed out from each data set together with motion translation and rotation estimates after segmenting the entire brain, the WM, and ventricles from the normalized T1 volume.

Four seed regions (right and left pre-central gyri, middle cerebellar peduncles, and pons) were defined from anatomical brain atlases "Talairach-labels-1 mm" and "John Hopkins University" in the Functional MRI of the Brain (FMRIB) Software Library (FSL) (Lancaster et al., 2007). To compute functional connectivity maps corresponding to a selected seed region of interest (ROI), the mean regional time course was extracted from all ROI voxels and correlated against all voxels of the brain. Separate correlation maps were produced for each subject in each group and ROI. The correlation maps were applied with the Fisher's transform z = 0.5 Ln [(1 + r)/(1 – r)] before entering a second-level random-effects statistical analysis where the main and differential effects of the two studied groups were summarized as t-statistic maps. This analysis was carried out by treating the individual subject map values as random observations at each voxel. Therefore, the classical analysis of variance (ANOVA) was performed at each voxel to map the whole-brain distribution of the seed-based functional connectivity for the difference between the two groups, using age and gender as nuisance covariates. To correct for multiple comparisons in the voxel-based analysis, regional effects resulting from the voxel-based comparative tests were only accepted for compact clusters surviving the joint application of a voxel- and cluster-level threshold chosen with a nonparametric randomization approach. Namely, an initial voxel-level threshold was set to *p* = 0.001 (uncorrected) and a minimum cluster size was estimated after 1,000 Monte Carlo simulations that protected against false-positive clusters up to 5% (Eklund et al., 2016; Forman et al., 1995).

Individual z-scores from regions identified in the above analysis were also extracted and used in linear correlation analysis in the ALS patients’ group with the PBA index (i.e., CNS-LS score) and ALSFRS-R bulbar subscore performed by the "corrcoef" function of MATLAB. For these regional analyses, we used the Pearson linear correlation coefficient and a statistical significance level of *p* ≤ 0.05 (Bonferroni corrected).

#### Regional atrophy measurements: voxel-based morphometry (VBM)

We performed a whole-brain VBM analysis using the SPM12 software package (http://www.fil.ion.ucl.ac.uk/spm/) with default parameters incorporating the DARTEL toolbox to obtain a high-dimensional normalization protocol (Ashburner, 2007). Images were bias-corrected, tissue-classified, and registered using a unified model with default parameters incorporating the DARTEL toolbox. Subsequently, the warped GM segments were affine transformed into MNI space and were scaled by the Jacobian determinants of the deformations to account for the local compression and stretching that occurs as a consequence of the warping and affine transformation (modulated GM volumes). Moreover, modulated images were smoothed with an 8-mm full-width half-maximum Gaussian kernel to create the final probability maps (Henley et al., 2010). An unpaired t-test was used to compare PBA and without PBA patients to HC. GM atrophy results of between-group comparisons were Family-Wise Error (FWE) corrected at a level of *p* < 0.05 and covaried for age, gender, and total intracranial volume (TIV; i.e., the sum of GM, WM, and CSF volumes) (Malone et al., 2015).

## Results

### Demographics and clinical variables

Patient and control characteristics are reported in Table 1. ALS patients and HC did not statistically differ in age, gender, and education. Considering that most subjects were men (about 85% of the patients’ groups), to minimize the possible influence of gender on the results, statistical analyses were implemented, considering gender as a covariate. A two-tailed t-test revealed no significant difference in global cognitive performance (i.e., the total score and subscores of ECAS) between PSB and nPSB patients. The two subsets were significantly different regarding ALSFRS-R bulbar subscore (*p* = 0.03) and CNS-LS score (*p* < 0.001), confirming this difference between the two groups in both laughing (*p* < 0.001) and crying (*p* = 0.003) scores. Moreover, major impairment of bulbar functions and pseudobulbar signs were revealed in the PSB group. On the base of ECAS subscores (Poletti et al., 2016; Siciliano et al., 2017), according to the Strong criteria for frontotemporal spectrum disorder of ALS (Strong et al., 2017), using the cut-off according to Siciliano et al. (2017), 9 patients had ALSci (i.e., 6 with executive function impairment and 3 with both executive and language impairments). In particular, 3 patients with executive dysfunction were revealed in both groups, and 2 patients and 1 patient with both executive and language impairment were reported, respectively, in the nPSB and PSB groups.

**Table 1:** Demographic, clinical, and neuropsychological measures of patients, divided into pseudobulbar (PSB) and non-pseudobulbar (nPSB), and healthy controls (HC); data are shown as mean (standard deviation) or count (percentage)

Variables	HC (*n* = 26)	ALS (*n* = 27)	PSB (*n* = 13)	nPSB (*n* = 14)	HC vs ALS p-value	PSB vs nPSB p-value
Age, years	58 (12)	58 (9)	56 (9)	60 (10)	0.947	0.271
Education, years	11 (4)	11 (4)	9 (3)	12 (5)	0.824	0.134
Sex, male	20 (76.9%)	23 (85.2%)	11 (84.6%)	12 (85.7%)	0.453	0.328
Disease Duration, months	n.a	21 (21)	16 (17)	25 (25)	n.a	0.24
Disease onset (bulbar/upper limbs/lower limbs)	n.a	5/10/12	4/5/4	1/5/8	n.a	0.157
Disease phenotype (classic/bulbar/FL/FA/pyramidal)	n.a	3/3/5/5/11	2/2/1/3/5	1/1/4/2/6	n.a	0.332
ALSFRS-R total score	n.a	43 (3)	42 (4)	43 (3)	n.a	0.773
Bulbar subscore	n.a	11 (1)	11 (1)	12 (1)	n.a	**0.031**
Fine motor subscore	n.a	10 (2)	10 (2)	10 (2)	n.a	0.599
Gross motor subscore	n.a	10 (2)	10 (2)	9 (2)	n.a	0.147
Respiratory subscore	n.a	12 (0.4)	12 (0.6)	12 (0)	n.a	0.221
UMN score	n.a	8 (4)	8 (4)	8 (5)	n.a	0.778
King’s clinical staging (1/2/3)*	n.a	11/13/3	5/5/3	6/8/0	n.a	0.305
MMSE	29(1)	n.a				
ECAS total score	n.a	88 (25)	83 (22)	92 (27)	n.a	0.326
Language	n.a	21 (5)	20 (4)	22 (5)	n.a	0.441
Verbal fluency	n.a	16 (7)	16 (7)	16 (8)	n.a	0.993
Executive functions	n.a	27 (11)	24 (11)	29 (11)	n.a	0.269
Memory	n.a	13 (5)	12 (5)	14 (5)	n.a	0.205
Visuospatial abilities	n.a	11 (1)	10 (1)	11 (1)	n.a	0.214
CNS-LS	n.a	12 (4)	16 (2)	9 (2)	n.a	** < 0.001**
Laughing	n.a	7 (3)	9 (3)	5 (1)	n.a	** < 0.001**
Crying	n.a	5 (2)	6 (2)	4 (1)	n.a	**0.003**
BDI-II	n.a	7 (6)	6 (6)	8 (7)	n.a	0.449

*ALS* Amyotrophic Lateral Sclerosis; *ALSFRS-R* ALS Functional Rating Scale Revised; *BDI-II* Beck Depression Inventory -II; *ECAS* Edinburgh Cognitive and Behavioural ALS Screen; *FA* flail arm; *FL* flail leg; *MMSE* Mini Mental State Examination; *HC* Healthy Controls; *χ2* Chi-square test; *UMN upper motor neuron*

^*^ *According to the King’s clinical staging system, the number of regions involved gives the stage*

### RSN functional connectivity: ICA

When comparing ALS patients to HC and PSB to nPSB patients, among RSNs, the sensorimotor network (SMN), the default mode network (DMN), the frontoparietal network (FPN), including the right and left components, and the salience network (SLN) components showed statistically significant regional between-group effects in their spatial distribution.

#### SMN

**Fig. 1:**
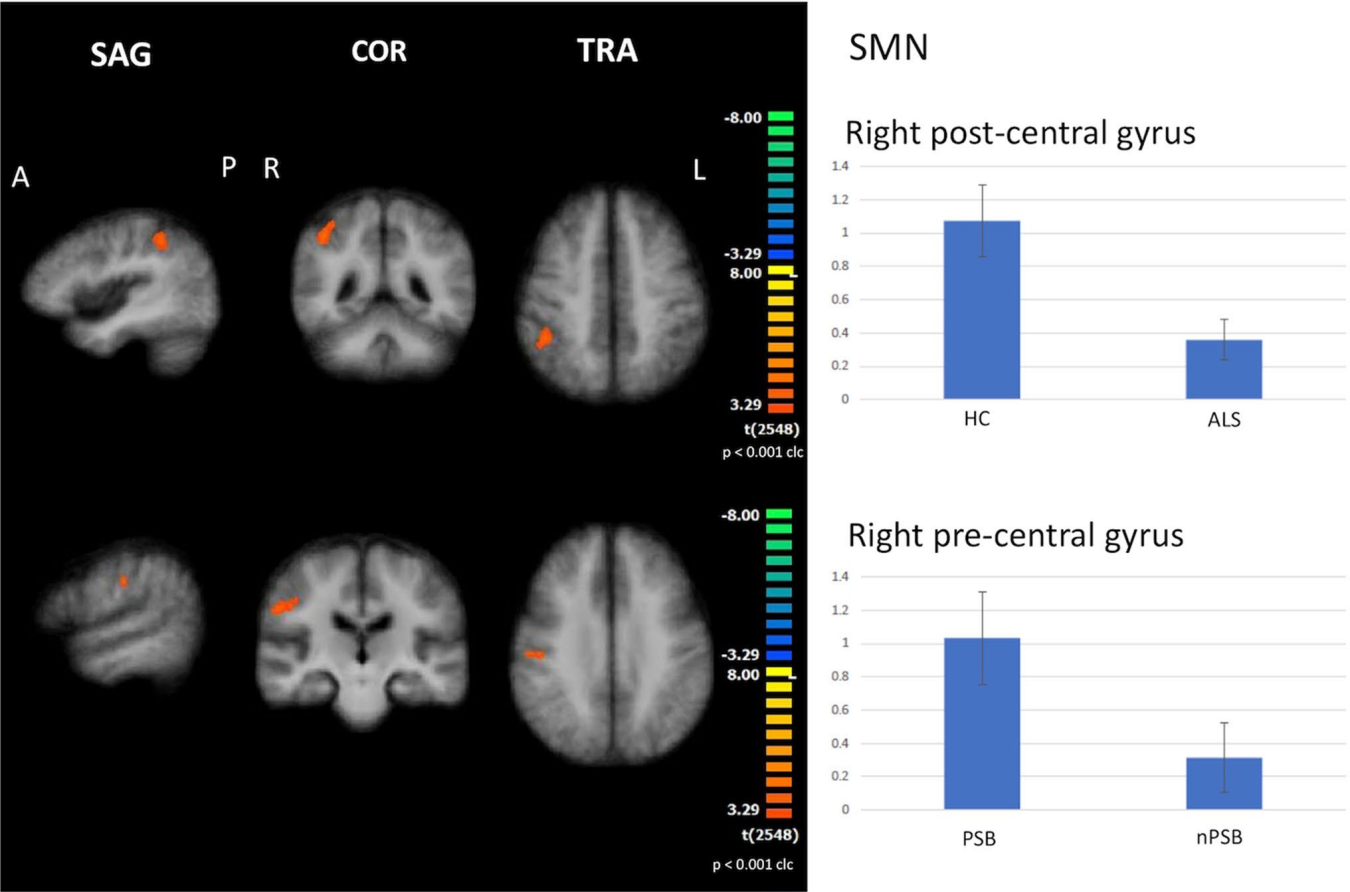
Results of ICA: SMN abnormalities Decreased functional connectivity in the right post-central gyrus (cluster-level corrected *p* ≤ 0.05, voxel-level *p* ≤ 0.001) by comparing all ALS patients to healthy controls (HC) (upper panels) and increased functional connectivity in the right pre-central gyrus by comparing pseudobulbar patients (PSB) to non-pseudobulbar (nPSB) patients (cluster-level corrected *p* ≤ 0.05, voxel-level *p* ≤ 0.001) (lower panels) (left: between-group comparisons maps, red-yellow scale; right: bar plots of the average functional connectivity levels). A = anterior; clc = cluster-level corrected; COR = coronal; ICA = independent-component analysis; L = left; P = posterior; R = right; SAG = sagittal; SMN = sensorimotor network; TRA = transverse

When compared to HC, all ALS patients exhibited decreased FC in the right post-central gyrus (cluster-level corrected *p* ≤ 0.05, voxel-level *p* ≤ 0.001), as well as observed in each subset of patients (i.e., PSB, nPSB) in comparison to HC (Fig. 1).

Between-subgroup comparison showed that PSB patients exhibited increased FC in the right pre-central gyrus in comparison to nPSB patients (cluster-level corrected *p* ≤ 0.05, voxel-level *p* ≤ 0.001) (Fig. 1).

#### DMN

**Fig. 2:**
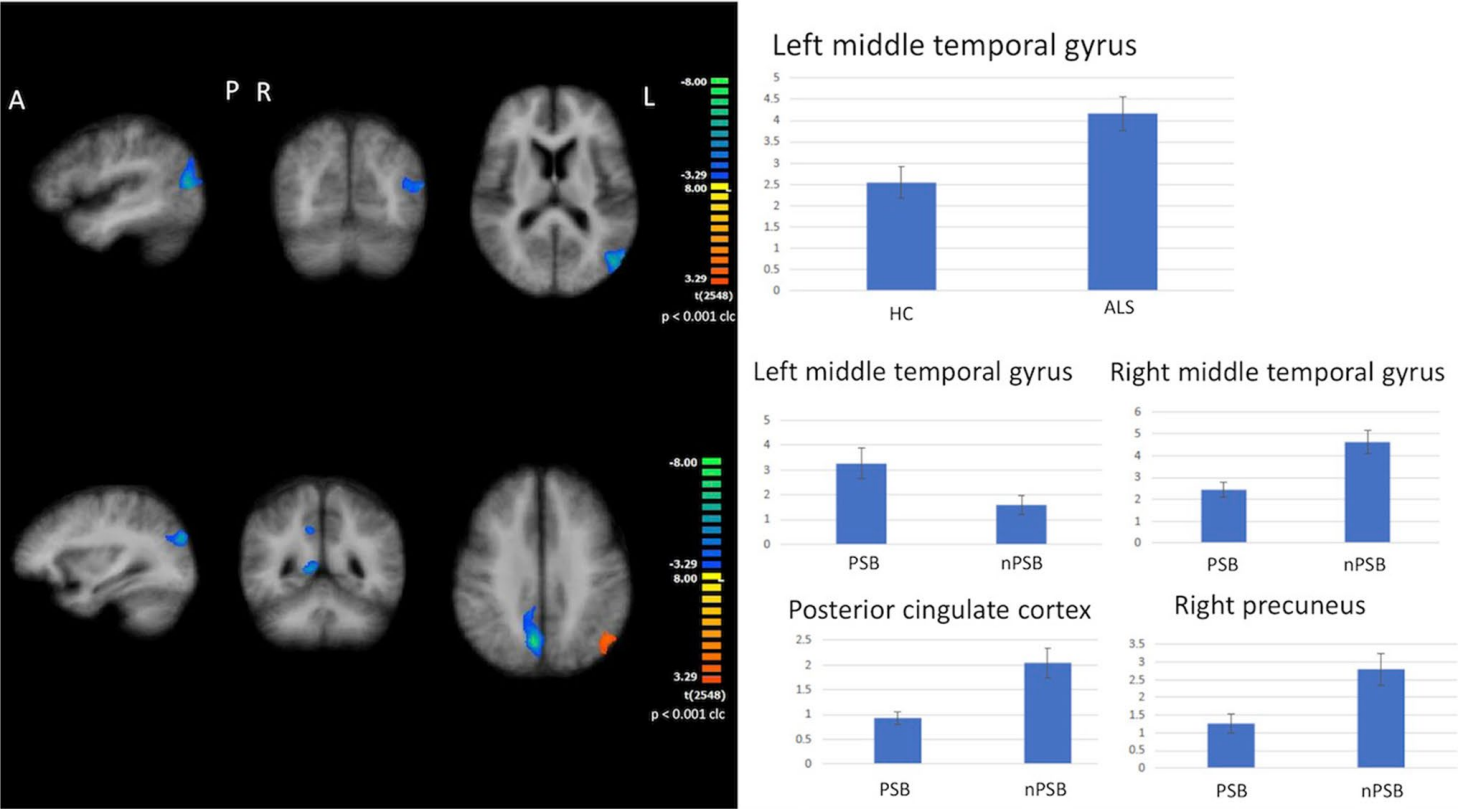
Results of ICA: DMN abnormalities Increased functional connectivity in the left middle temporal gyrus (cluster-level corrected *p* ≤ 0.05, voxel-level *p* ≤ 0.001) by comparing all ALS patients to HC (upper panels); increased functional connectivity in the left middle temporal gyrus and decreased functional connectivity in the right middle temporal gyrus, right precuneus and posterior cingulate cortex by comparing PSB patients to nPSB ones (cluster-level corrected *p* ≤ 0.05, voxel-level *p* ≤ 0.001; lower panels) (left: between-group comparisons maps, blue scale; right panels: bar plots of the average functional connectivity levels). A = anterior; clc = cluster-level corrected; COR = coronal; DMN = default mode network; ICA = independent-component analysis; L = left; P = posterior; R = right; SAG = sagittal; TRA = transverse

When compared to HC, all ALS patients exhibited increased FC in the left middle temporal gyrus (cluster-level corrected *p* < 0.05, voxel-level *p* < 0.001), as well as in both subsets of ALS patients who also showed a decreased FC in the left precuneus (Fig. 2).

Between-subgroup comparison showed that PSB patients exhibited an increase of FC in the left middle temporal gyrus and a decrease of FC in the right middle temporal gyrus, right precuneus, and posterior cingulate cortex compared to nPSB patients (cluster-level corrected *p* < 0.05, voxel-level *p* < 0.001) (Fig. 2).

#### FPN

**Fig. 3:**
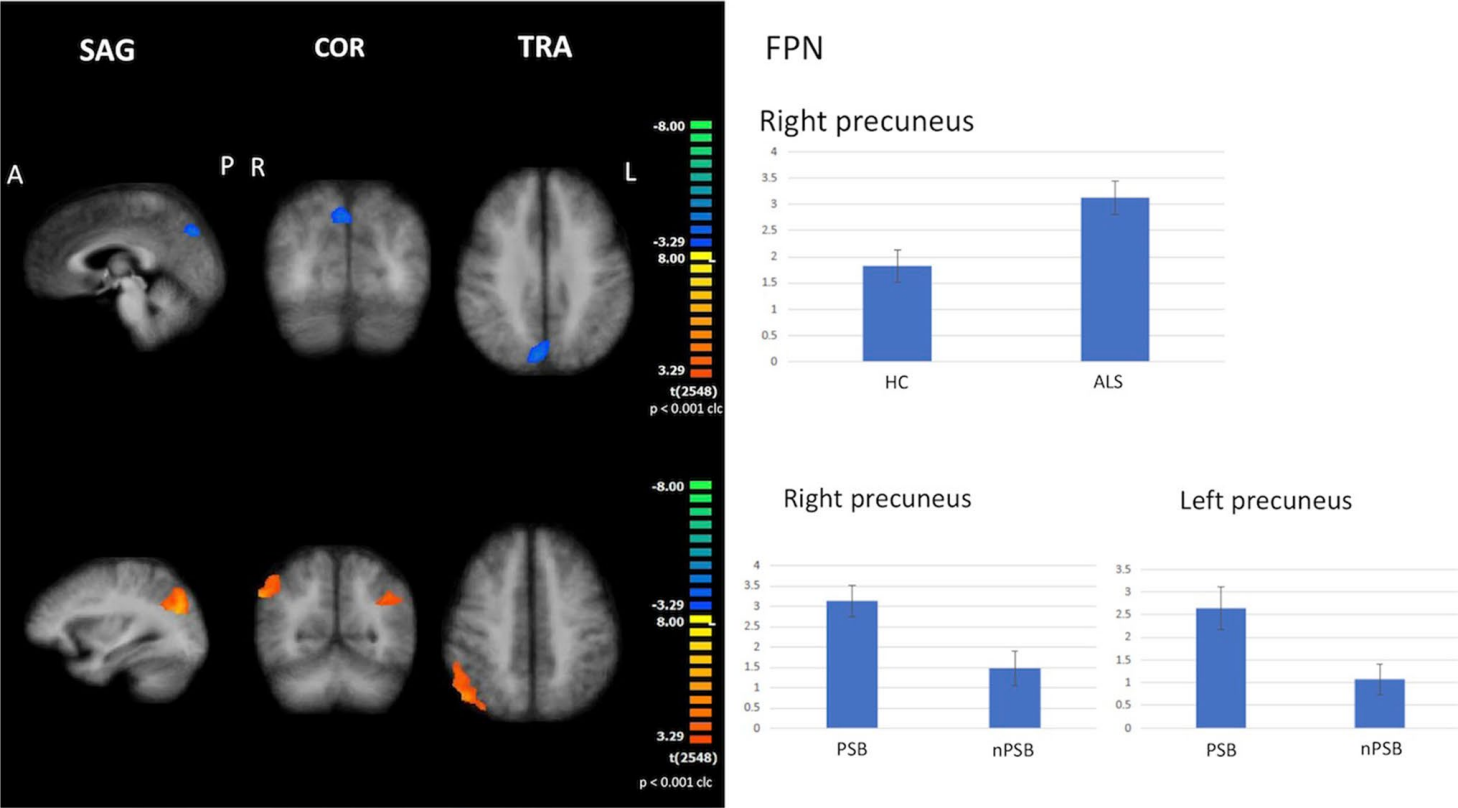
Results of ICA: FPN abnormalities Increased functional connectivity in the right precuneus (cluster-level corrected *p* ≤ 0.05, voxel-level *p* ≤ 0.001) by comparing all ALS patients to HC (upper panels); increased functional connectivity in the right and left precuneus by comparing PSB patients to nPSB ones (cluster-level corrected *p* ≤ 0.05, voxel-level *p* ≤ 0.001; lower panels) (left: between-group comparisons maps, blue/red-yellow scale; right: bar plots of the average functional connectivity levels). A = anterior; clc = cluster-level corrected; COR = coronal; FPN = frontoparietal network; ICA = independent-component analysis; L = left; P = posterior; R = right; SAG agittal; TRA = transverse

When compared to HC, all ALS patients exhibited increased FC in the right precuneus (cluster-level corrected *p* ≤ 0.05, voxel-level *p* ≤ 0.001) (Fig. 3). PSB patients showed increased FC in the right precuneus when compared to HC, while nPSB patients showed decreased FC in right and left superior occipital gyri when compared to HC (cluster-level corrected *p* ≤ 0.05, voxel-level *p* ≤ 0.001).

Between-subgroup comparisons showed that PSB patients exhibited increased FC in the right and left precuneus in comparison to nPSB patients (cluster-level corrected *p* ≤ 0.05, voxel-level *p* ≤ 0.001) (Fig. 3).

#### SLN

**Fig. 4:**
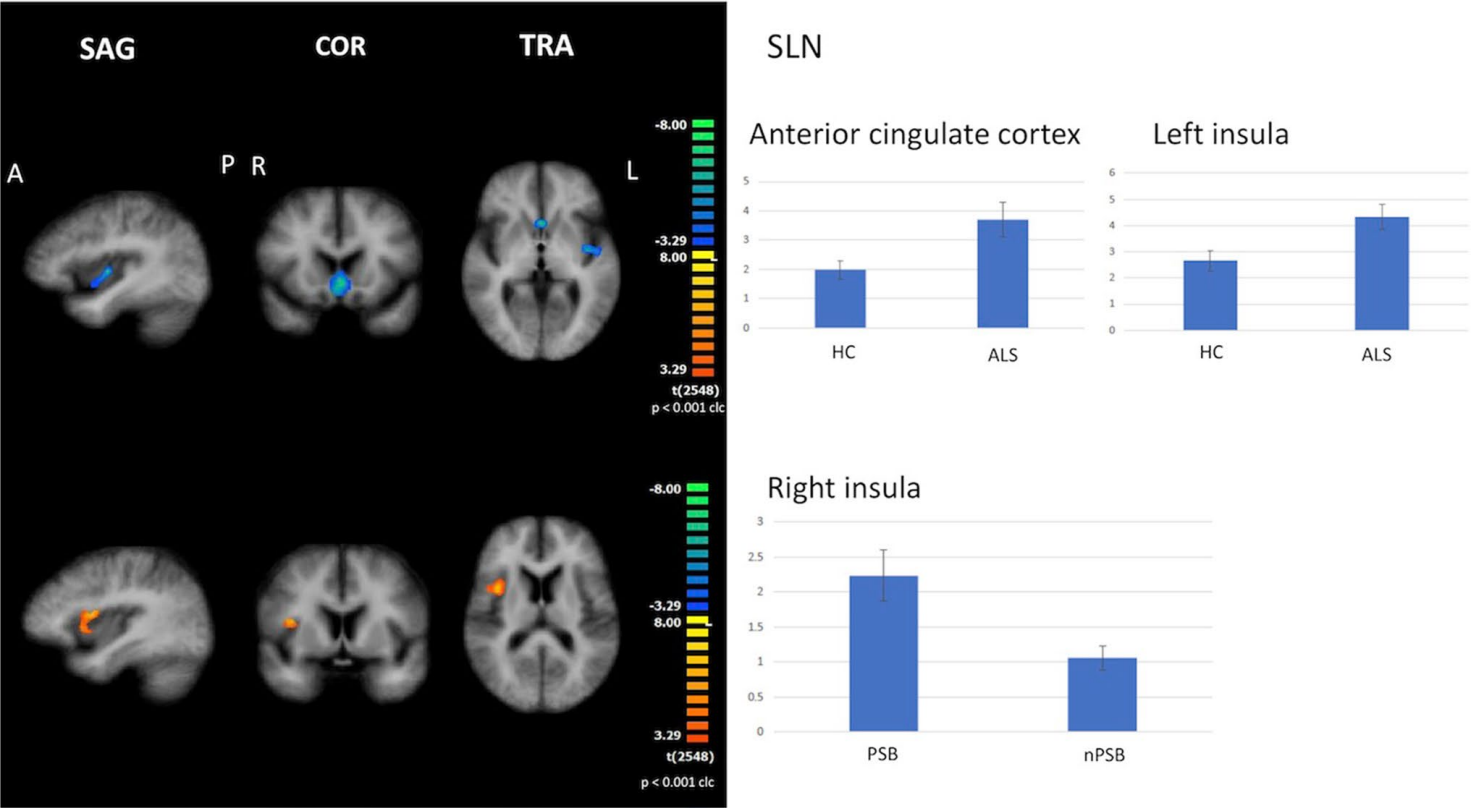
Results of ICA: SLN abnormalities Increased functional connectivity in the anterior cingulate cortex and left insula (cluster-level corrected *p* ≤ 0.05, voxel-level *p* ≤ 0.001) by comparing all ALS patients to HC (upper panels); increased functional connectivity in the right insula by comparing PSB patients to nPSB ones (cluster-level corrected *p* ≤ 0.05, voxel-level *p* ≤ 0.001: lower panels) (left: between-group comparisons maps, blue/red-yellow scale; right: bar plots of the average functional connectivity levels). A = anterior; clc = cluster-level corrected; COR = coronal; ICA = independent-component analysis; L = left; P = posterior; R = right; SAG agittal; SLN = salience network; TRA = transverse

When compared to HC, all ALS patients showed increased FC in the anterior cingulate cortex and left insula (cluster-level corrected *p* ≤ 0.05, voxel-level *p* ≤ 0.001), as well as in each subset of the patient when compared to HC (Fig. 4).

Between-subgroup comparison showed that PSB patients exhibited increased FC in the right insula in comparison to nPSB patients (cluster-level corrected *p* ≤ 0.05, voxel-level *p* ≤ 0.001) (Fig. 4).

#### Correlation analysis between CNS-LS and FC

With regard to SMN, FC in the right post-central gyrus (z-score) in the whole ALS group was found to be positively related to CNS-LS score (r = 0.4938, *p* = 0.0088). With regard to DMN, FC in the right middle temporal gyrus (z-score) in the whole ALS group was found to be inversely related to CNS-LS score (r = -0.4806, *p* = 0.0112). Regarding FPN, FC in the right and left precuneus (z-score) in the whole ALS group was found to be positively related to CNS-LS score (right precuneus r = 0.5259, *p* = 0.0048; left precuneus r = 0.4137, *p* = 0.0319). Finally, with regard to SLN, FC in the right insula and anterior cingulate cortex (z-score) in the whole ALS group was found positively related to CNS-LS score (right insula r = 0.4325, *p* = 0.0242; anterior cingulate cortex r = 0.7104, *p* = 0.0065). To note, no significant correlation was described between FC z-scores and ALSFRS-R bulbar score.

### RSN functional connectivity: seed-based analysis

**Fig. 5:**
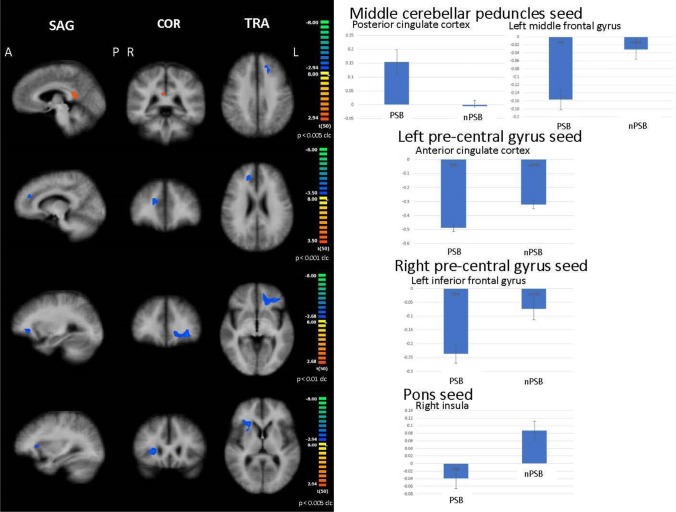
Results of seed-based connectivity analysis Abnormal functional connectivity between middle cerebellar peduncles (first panels), left and right pre-central gyrus (second and third panels), and pons (forth panels), as seeds, and other brain areas in PSB patients compared to nPSB ones (left: between-group comparisons maps, blue/red-yellow scale; right: bar plots of the average functional connectivity levels). A = anterior; clc = cluster-level corrected; COR = coronal; L = left; P = posterior; R = right; SAG = sagittal; TRA = transverse

Considering the middle cerebellar peduncles as seed, when compared to nPSB patients, FC was shown to decrease between bilateral middle cerebellar peduncles and the left middle frontal gyrus and increased between this seed and the posterior cingulate cortex in the PSB group (*p* ≤ 0.005 cluster-level corrected) (Fig. 5). No significant differences were shown by comparing all ALS patients to HC.

Considering left and right pre-central gyri as seeds, when compared to nPSB patients, decreased FC was found between the left pre-central gyrus and the anterior cingulate cortex (*p* ≤ 0.001 cluster-level corrected) and between the right pre-central cortex and the left inferior frontal gyrus (*p* ≤ 0.01 cluster-level corrected) in the PSB (Fig. 5). No significant differences were shown by comparing all ALS patients to HC.

Considering the pons as seed, when compared to nPSB patients, decreased FC was reported between the pons and the right insula (*p* ≤ 0.005 cluster-level corrected) in the PSB group. When compared to HC, decreased FC was found between the pons and the right middle frontal gyrus (*p* ≤ 0.005 cluster-level corrected) in the whole ALS group (Fig. 5).

### VBM analysis

No significant difference was revealed in GM comparing patients with PBA and those without PBA to HC and the two subsets of patients between each other (*p* < 0.05, FWE corrected).

## Discussion

In this study, we revealed RS-fMRI changes in several brain networks in a sample of ALS patients with PBA compared to ALS patients without PBA, underlying the major involvement of extra-motor fronto-temporo-parietal areas and of the connectivity between some fronto-temporal areas and middle cerebellar peduncles/pons in determining PBA in ALS. However, our findings showed FC abnormalities in both motor and extra-motor RSNs in the absence of whole-brain GM detected by VBM. Moreover, the seed-based analysis showed that FC between middle cerebellar peduncles and the right middle frontal gyrus, between the pons and the right insula, and between the left and right pre-central gyri and, respectively, the anterior cingulate cortex and the left inferior frontal gyrus was impaired in the PSB group compared to the nPSB group. Contrariwise, FC was increased between middle cerebellar peduncles and the posterior cingulate cortex in the PSB group compared to the nPSB one.

### RSN functional connectivity: ICA

The decrease of FC in the right middle temporal gyrus, right precuneus, and posterior cingulate cortex in the DMN shown in the group of ALS patients with PBA compared to nPSB patients recalled the involvement in PBA of brain areas, mainly in the right hemisphere, related to voluntary control of facial expression through excitatory projections to the gray periaqueductal (i.e., emotional system) (Klingbeil et al., 2021). Moreover, the decreased FC between the left and right pre-central gyri and, respectively, the anterior cingulate cortex and the left inferior frontal gyrus in the PSB group compared to the nPSB one may be related to the previously reported impairment of the "volitional system" descending from the motor and premotor cortices to inhibit laughter or crying (Klingbeil et al., 2021). Furthermore, the findings of increased FC in the right pre-central gyrus (SMN), in the left middle temporal gyrus (DMN), in the right and left precuneus (FPN), and in the right insula (SLN), together with the evidence of decreased FC between middle cerebellar peduncles and right middle frontal gyrus and between pons and the right insula, in PSB patients compared to nPSB subjects, were in favor of the hypothesis that PBA may reflect dysfunction of multiple sites of the "cortico-limbic-subcortico-thalamo-ponto-cerebellar network", shown to be entirely impaired in PBA (King & Reiss, 2013In particular, within FPN, FC is mainly decreased in the right precuneus in the nPSB ALS patients, while the whole group of ALS patients may show increased FC in this area when compared to HC, principally related to higher z-scores in this area in the PBS group.

### Correlation analysis between CNS-LS and FC

FC (mean z-scores) was revealed to be significantly related to CNS-LS score in several "hub" areas of the "cortico-limbic-subcortico-thalamo-ponto-cerebellar network" (King & Reiss, 2013), showing a positive correlation between CNS-LS score and FC in the right post-central gyrus (SMN), in the right and left precuneus (FPN), and in the right insula and anterior cingulate cortex (SLN). These positive correlations confirm the involvement of cortico-limbic areas in triggering PBA. Conversely, an inverse correlation was described between CNS-LS score and FC in the right middle temporal gyrus (DMN), suggesting that this area could show a modulatory role on emotion expression.

### RSN functional connectivity: seed-based analysis

Our results of decreased FC between the middle cerebellar peduncles and the right middle frontal gyrus and increased FC between the middle cerebellar peduncles and the posterior cingulate cortex were consistent with the findings by Christidi et al. (2018) who reported structural MRI abnormalities in limbic/associative and cortico-cerebellar tracts (i.e., white matter, WM abnormalities) in ALS patients with PBA compared to those without PBA. These WM pathways have been found to be involved in the control of emotional expression through an emotionally-driven involuntary pathway (i.e., emotional system – limbic/associative areas) that is controlled and/or inhibited by a consciously-driven voluntary pathway (i.e., volitional system – premotor, motor, and somatosensory cortices) (Parvizi et al., 2009). In particular, the control of emotional expression is modulated via the connections through the pons returning backward to the cortex through the cerebellum and the thalamus (Parvizi et al., 2001). Regarding abnormalities of cerebellum volume and of the cerebro-cerebellar pathway in ALS, patterns of focal cerebellar changes have been reported in whole-brain analyses (Bede et al., 2021; Christidi et al., 2018; Prell & Grosskreutz, 2013). Moreover, Bede et al. (2021) revealed that the observation of cerebellar abnormalities in a large population of ALS patients, genotyped for carrying intermediate-length repeat expansions in *ATXN2* or hexanucleotide repeat expansions in *C9orf72*, was not driven by the detection of intermediate-length alleles of *ATXN2*, showing cerebellar atrophy (involving the posterior lobes and the vermis) in the group carrying repeat expansions in *C9orf72* and a predominantly anterior lobar pathology in sporadic ALS patients. Moreover, regarding cerebro-cerebellar connectivity, Bede et al. (2021) revealed that fronto-ponto-cerebellar and parieto-ponto-cerebellar WM pathways were impaired in the ALS population studied. Although we did not use a tractography approach, our evidence from seed-based analysis (i.e., decreased FC between the middle cerebellar peduncles and the right middle frontal gyrus and between the pons and the right insula and increased FC between the middle cerebellar peduncles and the posterior cingulate cortex) underlined the potential impairment in ALS of the two cerebro-cerebellar circuits also found damaged by Bede et al. (2021) in a large cohort of ALS patients. These results all together confirm that the pathogenetic mechanisms underlying ALS may also have significant roles in the biology of the cerebellum, potentially explaining the connectivity and structural changes that have been observed. In this regard, genetic causes could have a significant role considering the recent observation that genetic risk alleles for ALS also include spinocerebellar ataxia-associated genes (i.e., *ATXN1*, *ATXN2*) (Tazelaar et al., 2020; van Rheenen et al., 2021).

### Limits

The strength of our study is the combination of neuropsychological scores from well-validated tools (i.e., ECAS, CNS-LS) and MRI data in a cohort of ALS patients. However, there are some shortcomings to be acknowledged, including some methodological issues, such as the relatively small sample size and the cross-sectional study design. Despite the small sample size, the population studied recalled the numbers of cohorts of patients with ALS investigated in previous neuroimaging analyses (Floeter et al., 2014; Hübers et al., 2016; Trojsi et al., 2021; Tu et al., 2021).

## Conclusions

Functional connectivity abnormalities in patients with ALS and PBA when compared to ALS patients without PBA may underline the functional impairment in the PSB subset of patients of the network underpinning the emotional and volitional systems, which are shown to regulate the expression of emotions. Our findings reinforce the concept of direct involvement of the cerebellum in the abnormal modulation of emotional expression in PSB patients with ALS through connections returning backward to the cortex via subcortical structures, including the cerebellum. Future longitudinal studies, performed in larger samples and using multi-modal MRI approaches, will be needed to better describe the extent and nature of impairment of brain circuits underpinning PBA in ALS and their response to symptomatic treatments of this syndrome.

## Data Availability

All data and materials support the reported claims and comply with standards of data transparency. Deidentified data will be shared on reasonable request with the corresponding author.
